# Nanocatalysis MoS_2_/rGO: An Efficient Electrocatalyst for the Hydrogen Evolution Reaction

**DOI:** 10.3390/molecules29020523

**Published:** 2024-01-20

**Authors:** Fernando Guzmán-Olivos, Lucas Patricio Hernández-Saravia, Ronald Nelson, Maria de los Angeles Perez, Francisco Villalobos

**Affiliations:** 1 Departamento de Física, Facultad de Ciencias, Universidad Católica del Norte, Avda. Angamos 0610, Antofagasta 1270709, Chile; mpp014@alumnos.ucn.cl (M.d.l.A.P.); francisco.villalobos@alumnos.ucn.cl (F.V.); 2 Instituto de Alta Investigación, Universidad de Tarapacá, Av. General Velazquez 1775, Arica 1010069, Chile; 3 Departamento de Química, Facultad de Ciencias, Universidad Católica del Norte, Avda. Angamos 0610, Antofagasta 1270709, Chile; rnelson@ucn.cl

**Keywords:** hydrogen evolution reaction, electrocatalysis, MoS_2_, reduced graphene oxide, MoS_2_/rGO(2)

## Abstract

In this study, a systematic investigation of MoS_2_ nanostructure growth on a SiO_2_ substrate was conducted using a two-stage process. Initially, a thin layer of Mo was grown through sputtering, followed by a sulfurization process employing the CVD technique. This two-stage process enables the control of diverse nanostructure formations of both MoS_2_ and MoO_3_ on SiO_2_ substrates, as well as the formation of bulk-like grain structures. Subsequently, the addition of reduced graphene oxide (rGO) was examined, resulting in MoS_2_/rGO(n), where graphene is uniformly deposited on the surface, exposing a higher number of active sites at the edges and consequently enhancing electroactivity in the HER. The influence of the synthesis time on the treated MoS_2_ and also MoS_2_/rGO(n) samples is evident in their excellent electrocatalytic performance with a low overpotential.

## 1. Introduction

The present state of humanity is genuinely concerning due to the predominant use of current fuels, specifically fossil fuels. Not only are these fuels the primary contributors to climate change through the release of carbon dioxide [1,2], but they also pose a direct threat to human health. Additionally, relying solely on these fuels is unsustainable in meeting the growing energy demands, impacting both industries and modern civilization. This situation poses a significant risk to the global economy and the well-being of humanity as a whole [3,4]. For these reasons, there is a growing interest in the search of cleaner fuels, with hydrogen emerging as the most promising alternative [5]. Several processes for the production of hydrogen have been explored, including photovoltaic, wind and electrochemical water splitting [5,6,7,8,9,10,11], the electrochemical one being the most striking alternative, due to the evolution of hydrogen (HER) [12,13,14].

In this context, although the division of water by HER plays a fundamental role in the generation of hydrogen from hydrogen, it implies that the electrocatalyst to be used must possess outstanding physicochemical properties, such as fast kinetics in electron and proton transfer, a large number of active sites, a low energy barrier in water division, rapid adsorption/desorption of hydrogen ions, high electrocatalytic performance and low overpotential [15,16,17]. However, the electrocatalysts with the aforementioned characteristics, such as Pt [18], Re [19], Pd [20], Ir [21], Rh [22] and Au [12], are noble metals that are both scarce and expensive, hindering their industrialization [23,24,25]. Therefore, researchers aiming for the industrialization of hydrogen fuel have shifted their focus to exploring new electrocatalysts made from more abundant materials, especially transition metals [26]. Among these, the most attractive options are transition-metal dichalcogenides (TMDs) such as WS_2_, ZrS_2_, MoSe_2_, ZrSe_2_, WSe_2_, ZrSSe and MoS_2_ [27,28,29,30,31]. The most striking of the dichalcogenides for HER applications is molybdenum disulfide (MoS_2_), since it offers high electrocatalytic properties due to its high number of active sites, a highly stable crystalline structure, and a mode of highly efficient mass transport to the active center, favoring HER applications [32]. These properties can be seen through theoretical calculations made by Nørskov et al., revealing that MoS_2_ ranks at the top of the “hydrogen adsorption volcano”. They reported a Gibbs free energy value in hydrogen adsorption (∆G_H_) of ~0.08 eV, which is comparable to the previously mentioned precious-metal-based electrocatalysts [16]. Due to its remarkable electrocatalytic properties, various MoS_2_ synthesis strategies have been proposed such as mechanical stripping [33], colloidal methods [34], liquid phase stripping [35], chemical intercalation [36], etching [37], electrodeposition [38,39], the heterogeneous structure method [40,41], green synthesis [42,43] and chemical vapor deposition (CVD) [44]. The heterogeneous structure synthesis method involves the growth of molybdenum disulfide on the substrate surface, with interactions with carbon-based nanomaterials [45]. This method holds significant promise for potential use in large-scale industrial hydrogen production. In this context, several research groups have actively been searching for new materials with electrocatalysts properties [46,47,48,49,50,51].

Therefore, this work differs from the traditional methods of MoS_2_ synthesis previously described, particularly from CVD methods that typically involve MoO_3_ powders, which do not allow control over the homogeneity of MoS_2_ deposition. In this study, a systematic investigation of MoS_2_ nanostructure growth on a SiO_2_ substrate was conducted using a two-stage process. Initially, a thin layer of Mo was grown through sputtering, followed by a sulfurization process employing the CVD technique. This two-stage process enables the control of diverse nanostructure formations of both MoS_2_ and MoO_3_ on SiO_2_ substrates, as well as the formation of bulk-like grain structures. Subsequently, the addition of reduced graphene oxide (rGO) was examined, resulting in MoS_2_/rGO(n), where graphene is uniformly deposited on the surface, exposing a higher number of active sites at the edges and consequently enhancing electroactivity in the HER. The influence of the synthesis time on the treated MoS_2_ and also on MoS_2_/rGO(n) samples is evident in their excellent electrocatalytic performance with a low overpotential.

## 2. Results and Discussion

### 2.1. Materials’ Characterization by SEM, EDX, XPS and Raman

**Figure 1:**
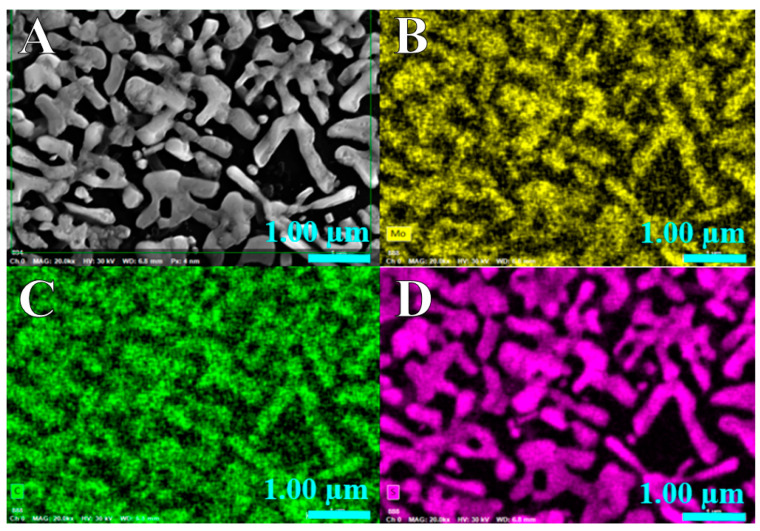
(**A**) SEM image and EDS element mapping of (**B**) Mo, (**C**) C and (**D**) S signal in MoS_2_/rGO(2).

The FE-SEM images in Figure 1 show the formation of elongated microstructures of MoS_2_ corresponding to the CVD-treated sample derived from a Mo thin film deposited for 25 min using sputtering. The color images correspond to EDS mappings of Mo, S and C in the micrographs. It is noticeable that the structures are primarily composed of S and Mo, represented by the highlighted colors in Figure 1. Additional information regarding the SEM images is provided in the Supplementary Material in Figures S1 and S2. From these figures, it can be observed that as the deposition time of the Mo thin film exposed to CVD thermal treatment increases, the type of formed structures changes. In Figure S1, for the thermal treatment carried out on the Mo thin film deposited for 15 min, only island-like structures are formed, whereas with an increase in the deposition time in sputtering, and consequently, the thickness of the Mo film, larger structures in the form of disks or bars are observed; the size of the MoS_2_ nanoparticles can be seen in Table S1. As shown in Figure S2, EDS mappings are presented alongside the SEM images. In these mappings, sulfur is detected in the structures for each case; however, this could be attributed to false readings due to overlap with the molybdenum peak. Subsequent Raman and XPS analyses clarify this effect.

XRD is an important characterization method for reflecting the composition and crystal structure of synthesized materials. Figure S3 shows the XRD of MoS_2_ heterostructures. The peaks at 14.4°, 33.00° and 61.7°, correspond to the (002), (101) and (110) 2H MoS_2_ phase planes (JCPDS card n°. 37-1492).

**Figure 2:**
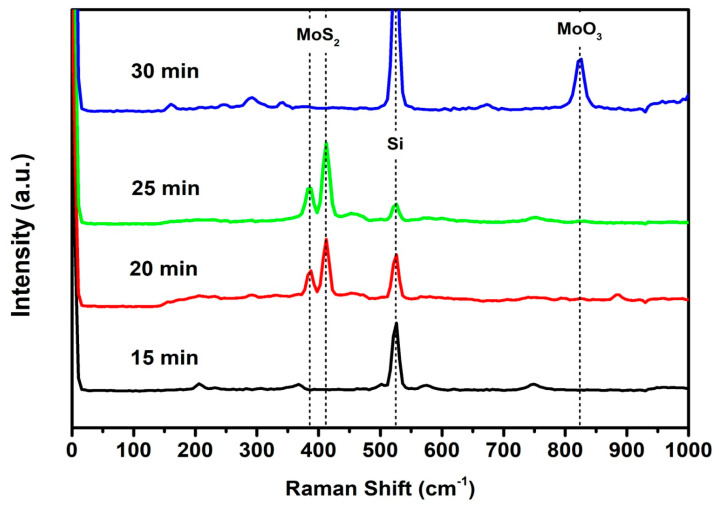
Raman spectra of various MoS_2_ samples.

The Raman spectra were obtained for MoS_2_ growth at several deposition times. Figure 2 show the characteristic peaks of MoS_2_ appearing at 387 and 412 cm^−1^, observed in the sample at 25 min. The separation between those peaks (~25 cm^−1^) suggests a bulk-like structure [52]. In the sample deposited for 30 min, a characteristic band of MoO_3_ is observed at 823 cm^−1^, corresponding to the M=O stretch mode [53] for MoO_3_. In the case of the sample deposited for 15 min, the presence of MoO_3_ or MoS_2_ is not observed, indicating either a very thin deposition or the absence of material.

**Figure 3:**
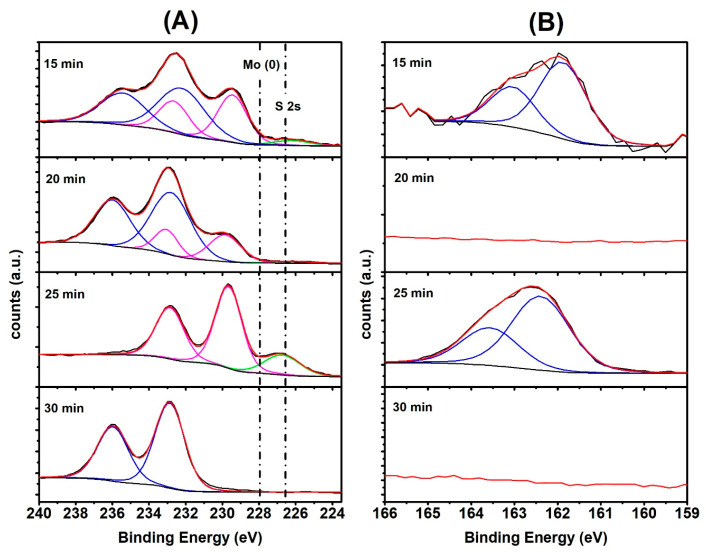
XPS synthesis of MoS_2_/rGO(n) at 15, 20, 25 and 30 min (**A**,**B**). Magenta lines of group A correspond to Mo(IV) and the blue ones are Mo(VI) (**A**). High-resolution XPS peak-shape of Mo 3d and S 2p, respectively.

In Supplementary Material Figure S4, a survey spectrum of the growth samples is presented. In these spectra, notable peaks include Mo 3d and Mo 3p, S 2p, corresponding to MoS_2_, along with Si 2p, Si 2s and O 1s peaks associated with the SiO_2_ substrate. Finally, the C 1s peak attributed to adventitious carbon and rGO is observed. XPS high-resolution spectra of the Mo 3d region are show in Figure 3A. The spectra of sample growth at 25 min showed the Mo(IV) oxidation state (magenta) at 229.5 eV and its doublet at 232.7 eV of binding energy. The presence of the S 2s state at 227 eV (green), characteristic of MoS_2_, is also observed. The metal Mo 3d5/2–3/2 peak typically appears at 227.8 eV with a splitting of ~3.13 eV. For samples grown for 15 and 30 min, the Mo(VI) oxidation state (blue) was observed at a binding energy of 233.2 eV [54]. In the XPS high-resolution spectra of the S 2p region in Figure 3B, a shift in the S 2p region was found at 163.9 eV [55] for the sample grown for 25 min.

In the 15 min sample, the observed shift corresponds to the characteristic sulfur transition in a non-stoichiometric molybdenum sulfide compound. In the 30 min sample, no sulfur presence was observed, corroborating the findings of the micro-Raman spectra, which only detect the characteristic transition of Mo(VI) species at 233.1 eV.

### 2.2. Electrochemical Characterization

**Figure 4:**
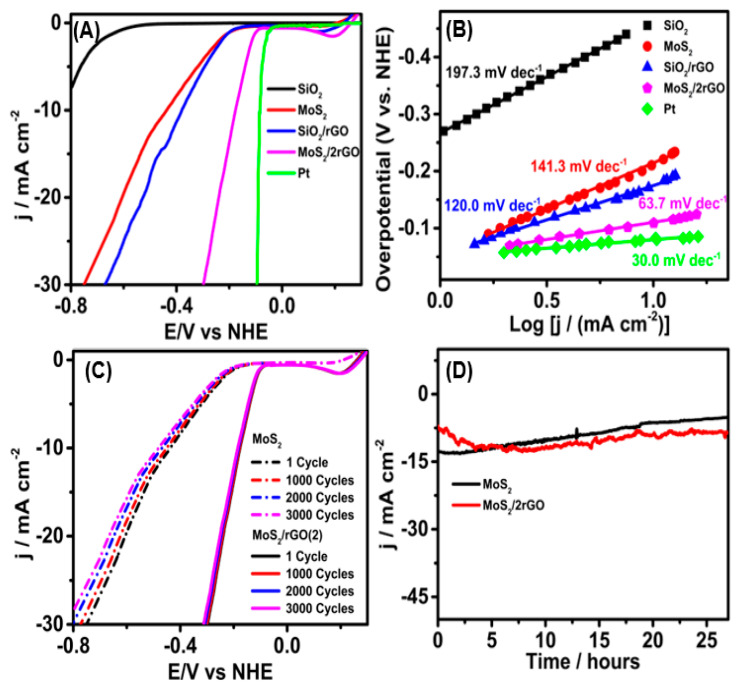
HER performance for LSV polarization curves recorded in 0.5 M H_2_SO_4_ solution with SiO_2_ (black), MoS_2_ (red), SiO_2_/rGO (blue), Pt/C (green) and MoS_2_/rGO(2) (magenta) electrodes at the scan rate of 2 mV s^−1^ (**A**) and corresponding Tafel plots (**B**). Durability measurement of the MoS_2_ and MoS_2_/rGO(2) in 0.5 M H_2_SO_4_ solution after 1000, 2000 and 3000 cycles (**C**) and chronoamperometric curves of at applied potentials of 447 and 176 mV, respectively, for 27 h (**D**).

To determine the optimal growth time of MoS_2_ on the SiO_2_ surface with prominent electrocatalytic properties for HER applications, a study of the catalytic properties of the MoS_2_ synthesized for 0, 15, 20, 25 and 30 min was carried out by means of the LSV technique in 0.5 M H_2_SO_4_ with a scan rate of 2 mV s^−1^ (Figure S5). The study revealed overpotentials (measured at 5 mA cm^−2^) of 0.761, 0.533, 0.423, 0.320 and 0.421 vs. RHE for 0, 15, 20, 25 and 30 min, respectively. Therefore, based on the polarization curves and supported by the XPS in Figure 3, we can conclude that the optimal synthesis time for MoS_2_ was 25 min. At 15 and 20 min, there is a mixture of Mo(IV) and Mo(VI), and after 30 min, it begins to resemble MoO_3_ (see Figure S4). On the other hand, the influence of rGO to improve the catalytic properties of MoS_2_ was explored; On the other hand, the influence of rGO to improve the catalytic properties of MoS_2_ was explored; as was recently reported, due to the strong van der Waals, the electrostatic interactions between rGO and MoS_2_ are strong [56,57]. In this sense, MoS_2_ (synthesized for 25 min) was doped with different concentrations of rGO (1, 2 and 4 mg L^−1^), as seen in Figure S6. The correlation in the improvement of water reduction is evident in the LSV curves shown in Figure S5. The best performance for the HER was achieved with the MoS_2_/rGO(2) electrode instead of the MoS_2_/rGO(4). This could be attributed to the aggregation caused by the π–π interactions between the layers of rGO [58]. This effect decreases the active sites of MoS_2_ and causes MoS_2_/rGO(4) to require a higher overpotential than MoS_2_/rGO(2). Figure 4A depicts the LSV curve of the as-obtained Pt/C, bare SiO_2_, MoS_2_, SiO_2_/rGO and MoS_2_/rGO(2) at a scan rate of 2 mV s^−1^. As it can be seen, the MoS_2_/rGO(2) electrode exhibited excellent electrocatalytic activity for HER compared to that of SiO_2_, MoS_2_ and SiO_2_/rGO electrodes. Accordingly, a shift in the onset potential towards a less negative potential value and a benchmark current density of 10 mA cm^−2^ at a very low overpotential (−0.176 V) can be noticed. An important aspect is to see by which mechanism it produces hydrogen, in which the adsorption of hydrogen is vital [59], and why the HER mechanism exists and whether it is Volmer–Tafel or Volmer–Heyrovsky, considering these Tafel plots were carried out. The catalytic activity of all the synthesized electrocatalysts was further investigated and studied via Tafel slope, as presented in Figure 4B. The Tafel slope was calculated using the observed LSV curves. The HER pathways and related kinetics were identified using the Tafel plots, generated with the equation overpotential (η) = a + b log |j|, where b is the Tafel slope, a is the intercept, η is the overpotential and j is the current density [60]. The long-term durability of the MoS_2_/rGO(2) electrode was tested by recording LSV polarization curves before and after 1000, 2000 and 3000 consecutive potential cycles (scan rate 2 mV s^−1^), and by applying a constant current of 15 mA cm^−2^ for 27 h. The interaction between the nanostructures (interstitial S atoms) and rGO improves its stability and catalytic properties. Significant changes in the LSV were observed in MoS_2_; however, this did not occur in MoS_2_/rGO(2) (Figure 4C), as it was also evident in the chronopotentiometric curve (Figure 4D). This suggests that the MoS_2_/rGO(2) presents efficient catalyst durability for HER applications, indicating that the interaction between MoS_2_ and rGO improves its catalytic properties and provides greater stability. The overpotentials and Tafel slopes of the above-described materials were compared with those of other catalysts recently described in the literature (Table 1). The MoS_2_ derivatives presented good electrochemical parameters, but MoS_2_/rGO(2) exhibited the best ones, and is expected to outperform all the others for HER.

## 3. Experimental Section

### 3.1. Chemicals and Apparatus

Sulfuric acid (H_2_SO_4_), molybdenum 99.95% (Mo), sulfur powder (S, 99%), reduced graphene oxide (rGO) and argon (Ar) were received from Merck (Rahway, NJ, USA) and Lesker (Hastings, UK). All solutions were prepared with Milli-Q water (18 MΩ cm). The working electrode (WE) was used SiO_2_, MoS_2_ or MoS_2_/rGO, a silver/silver-chloride (KCl sat.) electrode served as the reference electrode (RE), and a platinum wire was used as the counter electrode (CE). A Solartron model analytical potentiostat with Corrware software 2.0 was used for all electrochemical measurements. In this work, all the potentials were reported (E(RHE) = E(Ag/AgCl) + 0.197 V) vs. reversible hydrogen electrode (RHE).

### 3.2. Materials Synthesis

The growth of MoS_2_/rGO(n) nanostructures was conducted in a two-stage procedure. Initially, metallic molybdenum (Mo 99.95%) was deposited using sputtering on a 285 nm of thermal silicon dioxide for different synthesis times (15, 20, 25 and 30 min). Then, a treatment was performed using chemical vapor deposition (CVD) at 700 °C for 20 min. For this, 10 mg of sulfur powder was placed at the furnace entrance, and a mixture of Ar/H_2_ gases in a 4:1 ratio was introduced. The distance between molybdenum and sulfur was kept at 10 cm. After the reaction, the oven was cooled to room temperature. To determine the optimal synthesis time for MoS_2_, 30 µL of aqueous reduced graphene oxide (rGO) was added via drop casting at different concentrations (1, 2 and 4 mg mL^−1^) to obtain MoS_2_/rGO(1), MoS_2_/rGO(2) and MoS_2_/rGO(4), in order to observe the influence of rGO on the catalytic properties of MoS_2_.

### 3.3. Materials Characterization

For the Raman spectroscopy characterizations, a Witech Alpha300 micro-Raman system (Oxford Instruments, Abingdon, UK) was used, employing a 514 nm laser with a 2 mm spot size and a power of 2 mW. XPS spectra were obtained using a Surface Analysis Station 1, model XPS RQ300/2 equipped with a DESA150 detector/2700 V (STAIB Instruments, Minneapolis, MN, USA), with aluminum X-ray radiation of 1486.6 eV. The shift due to charging of the XPS spectra was corrected using the characteristic peak of adventitious carbon (C 1s) at its position of 284.8 eV. The spectra were analyzed using Casa XPS software (version 2.3.25), and for fitting, a 70% Gaussian/30% Lorentzian symmetric line-shape was employed. High-resolution electron microscopy analyses were performed using the SU500 Hitachi equipment (Hitachi, Tokyo, Japan). The surface morphology of the MoS_2_ (20, 25 and 30 min) and MoS_2_/nGO was investigated using a FE-SEM, Hitachi Brand, model SU5000, equipped with XFlash 6I30 detectors (Bruker brand, Billerica, MA, USA). The composition and distribution of the MoS_2_ (20, 25 and 30 min) and MoS_2_/GO(n) were ascertained by performing elemental analysis using an EDX instrument.

## 4. Conclusions

A two-stage method was proposed for synthesizing MoS_2_ nanostructures on the surface of a bare SiO_2_ electrode, involving sputtering and chemical vapor deposition. The chemical composition of MoS_2_ was controlled by maintaining a constant temperature of 700 °C during the thermal treatment, and the formation of nanostructures was explored using different thicknesses of molybdenum film. The two-stage method proposes an improvement in controlling the type of MoS_2_ nanostructures and the formation of bulk-like grains, according to Raman spectroscopy analysis. Also, XPS spectra reveal the presence of oxidation states corresponding to Mo(IV), and the shift in the peaks of the S 2p orbital confirms the formation of MoS_2_. Subsequently, the synthesized MoS_2_ was functionalized with rGO, and MoS_2_/rGO(2) demonstrated the best electrocatalytic performance. MoS_2_/rGO(2) exhibited enhanced electrocatalytic activity with a smaller Tafel slope of 63.7 mV dec^−1^ and a more positive onset overpotential of 176 mV, making it a promising electrocatalyst for HER in practical applications. The HER was observed to be strongly modulated by the heteroatom nature and content within the graphenic structure, as well as the interaction with MoS_2_. These results reveal the best performance for the HER in acidic media for MoS_2_/rGO(2). This is attributed to a precise amount of interstitial S atoms between graphene layers, which facilitates an increase in electron transfer. Additionally, the strong interaction between MoS_2_ and rGO contributes to an expansion degree of the S–Mo–S bonds. The HER activity was improved by adjusting the ratio of MoS_2_ and rGO. These results indicate that the MoS_2_/rGO electrocatalyst is a promising and cost-effective alternative for manufacturing electrodes, offering a potential solution for industrial-level hydrogen production.

## Figures and Tables

**Table 1:** Comparison of HER performances non-noble metals.

Material	Overpotential η_10_ (mV)	Tafel Slope (mV/dec)	Ref.
MoS_2_/rGO(2)	176	63.7	This work
MoS_2_	447	141.3	This work
Fe–Co–CN/rGO-700	213	97	[61]
Co_2_B NPs	328	92.4	[62]
NiS_2_/MoS_2_ HNW	204	65	[63]
NiO/rGO/NF	268	100	[64]
NiSx-WO2.9/NF	220	66	[65]
WP nanoparticles	254	65	[66]
S-gCN/NiV	560	79	[67]

## Data Availability

Data are contained within the article and Supplementary Materials.

## Supplementary Materials

The following supporting information can be downloaded at: https://www.mdpi.com/article/10.3390/molecules29020523/s1, Figure S1: SEM images for MoS_2_ samples grown through the thermal treatment of Mo thin films deposited via sputtering for 15 min (A), 20 min (E), 25 min (I) and 30 min (M). High magnification images are shown for MoS_2_ surfaces prepared on Mo thin films deposited via sputtering for 15 min (A–D), 20 min (E–H), 25 min (I–L) and 30 min (M–P); Figure S2: SEM images for the MoS_2_ samples grown via thermal treatment of Mo thin films deposited by sputtering for (A) 15, (D) 20, (G) 25, and (J) 30 minutes and EDS element mapping of Mo (B, E, H and K) and S (C, F, I and L) for each respective sample; Figure S3: X-ray diffraction patterns of MoS_2_; Figure S4: Survey spectra for the samples after the CVD process. The highlighted regions correspond to the different spectral peaks of the elements involved in the CVD process, Mo, Si, O and S; Figure S5: The linear sweep voltammograms (LSV) test at various synthesis time of MoS_2_ in 0.5 M H_2_SO_4_ solution 0 (black line), 15 (red line), 20 (blue line), 25 (pink line) and 30 minute (dark green line). Scan rate: 2 mV s^−1^; Figure S6: The HER LSV with MoS_2_/nrGO modified electrodes prepared with composites containing different proportions rGO, SiO_2_ (black line), SiO_2_/rGO (red line), MoS_2_/rGO (1) (blue line), MoS_2_/rGO(2) (pink line) and MoS_2_/rGO(4) (blue line). Scan rate: 2 mV s^−1^; Table S1: The size of MoS_2_ nanoparticles.
