# Supplementary material for: Nanocatalysis MoS2/rGO: An Efficient Electrocatalyst for the Hydrogen Evolution Reaction
Source: Molecules. 2024 Jan 20;29(2):523. doi: 10.3390/molecules29020523 (PMC10819749; doi:10.3390/molecules29020523)
Supplement: Supplementary file 1 [file molecules-29-00523-s001.zip › molecules-2805668-supplementary.pdf]

Supporting Information

Nanocatalysis MoS_2/rGO: An efficient electrocatalysts for the HER

Fernando Guzmán-Olivos[1]*, Lucas Patricio Hernández-Saravia[2]*, Ronald Nelson[3], Maria de los Angeles Perez[1] & Francisco Villalobos[1]

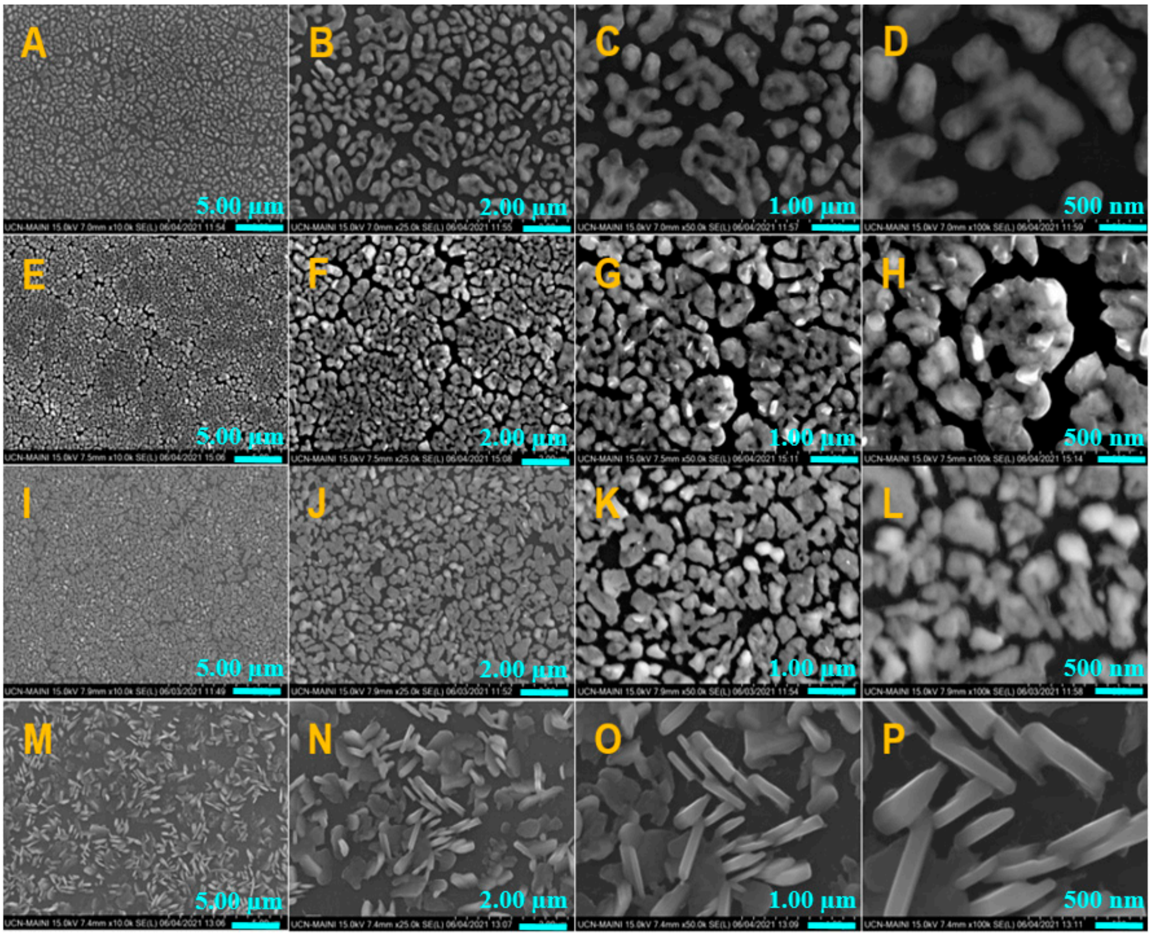

Figure S1: SEM images for MoS_2 samples grown through the thermal treatment of Mo thin films deposited via sputtering for 15 min (A), 20 min (E), 25 min (I) and 30 min (M). High magnification images are shown for MoS_2 surfaces prepared on Mo thin films deposited via sputtering for 15 min (A-D), 20 min (E-H), 25 min (I-L) and 30 min (M-P).

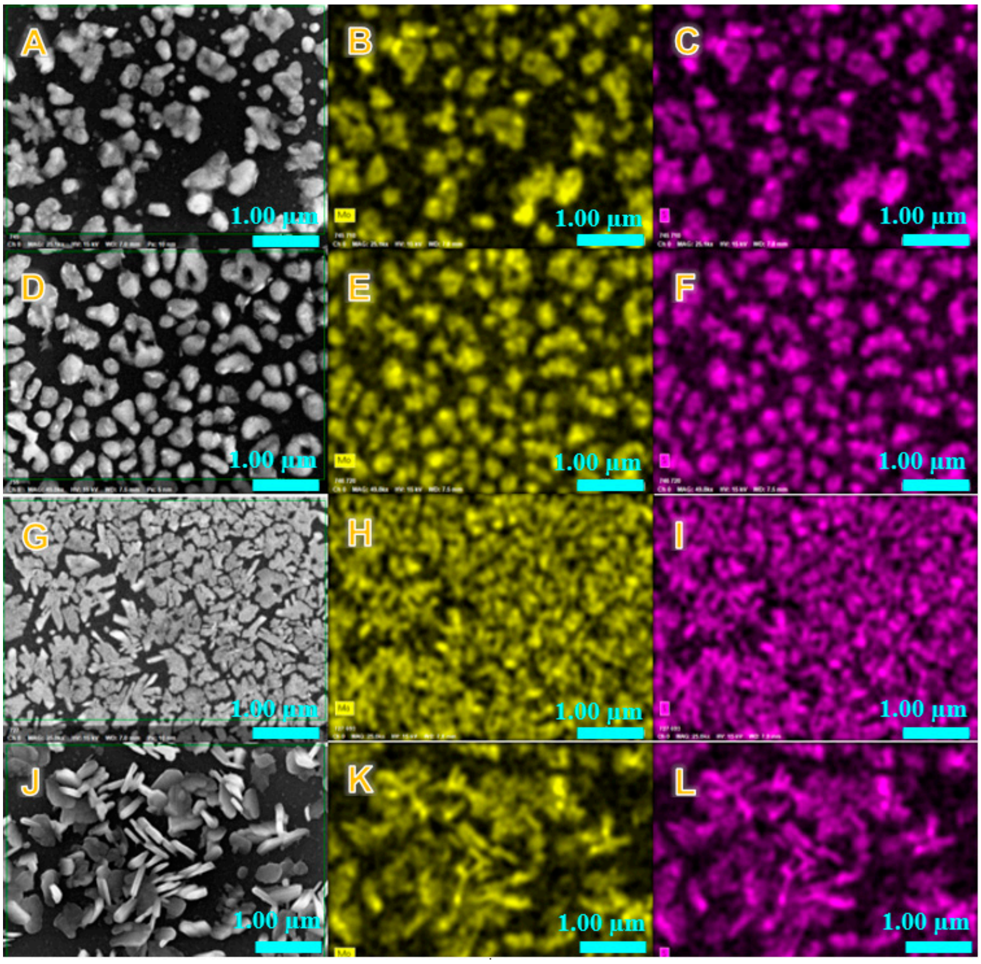

Figure S2: SEM images for the MoS_2 samples grown via thermal treatment of Mo thin films deposited by sputtering for (A) 15, (D) 20, (G) 25, and (J) 30 minutes and EDS element mapping of Mo (B, E, H and K) and S (C, F, I and L) for each respective sample.

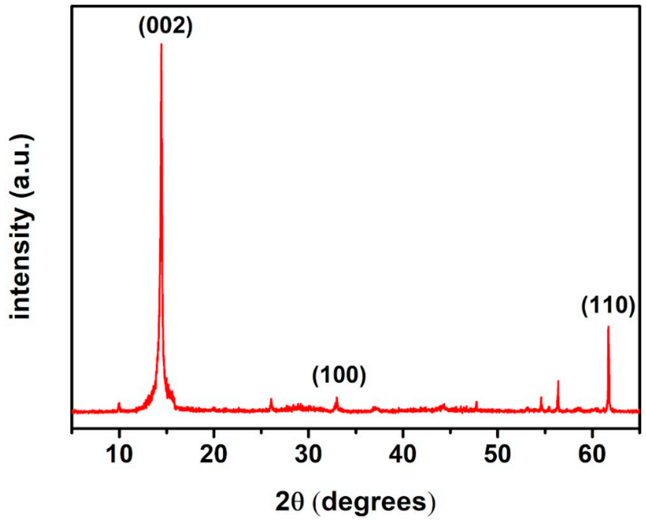

Figure S3: X-ray diffraction patterns of MoS_2

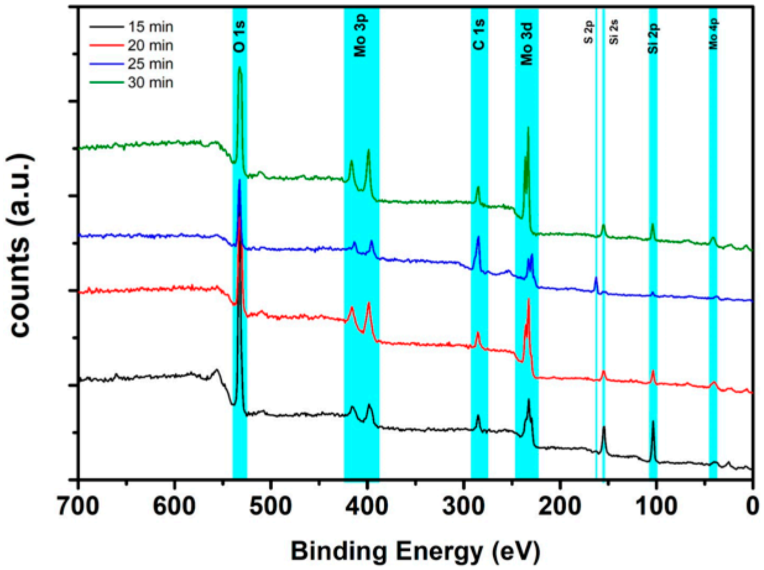

Figure S4: Survey spectra for the samples after the CVD process. The highlighted regions correspond to the different spectral peaks of the elements involved in the CVD process, Mo, Si, O and S

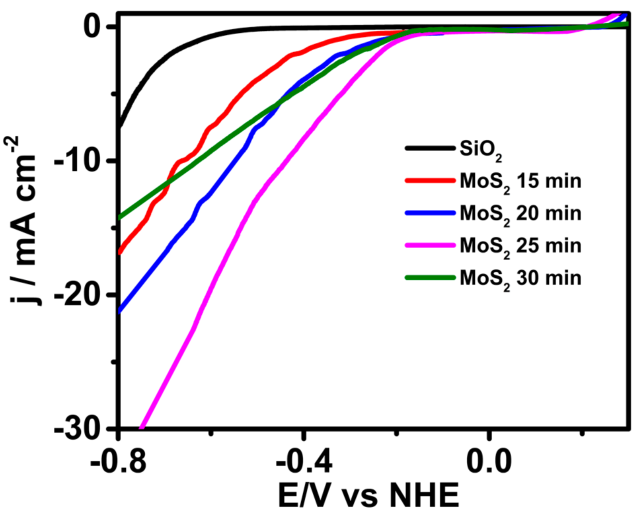

Figure S5: The linear sweep voltammograms (LSV) test at various synthesis time of MoS_2 in 0.5 M H_2SO_4 solution 0 (black line), 15 (red line), 20 (blue line), 25 (pink line) and 30 minute (dark green line). Scan rate: 2 mV s^{-1}.

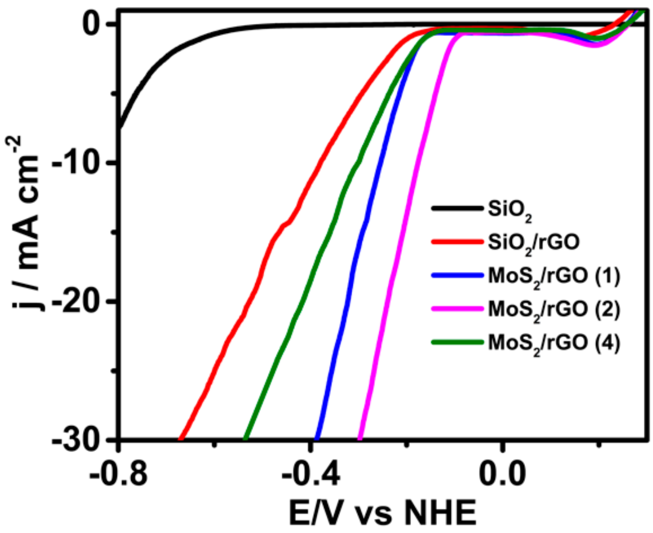

Figure S6: The HER LSV with MoS_2/nrGO modified electrodes prepared with composites containing different proportions rGO, SiO_2 (black line), SiO_2/rGO (red line), MoS_2/rGO (1) (blue line), MoS_2/rGO(2) (pink line) and MoS_2/rGO(4) (blue line). Scan rate: 2 mV s^{-1}.

Table S1: The size of MoS_2 nanoparticles

Synthesis time	Size (nm)
15	21.2
20	50.0
25	76.6
30	81.9
